# Effects of Protein-Iron Complex Concentrate Supplementation on Iron Metabolism, Oxidative and Immune Status in Preweaning Calves

**DOI:** 10.3390/ijms18071501

**Published:** 2017-07-12

**Authors:** Robert Kupczyński, Michał Bednarski, Kinga Śpitalniak, Krystyna Pogoda-Sewerniak

**Affiliations:** 1 Department of Environment Hygiene and Animal Welfare, Faculty of Biology and Animal Science, Wroclaw University of Environmental and Life Sciences, Chelmonskiego 38c, 51-630 Wroclaw, Poland; kinga.spitalniak@upwr.edu.pl (K.Ś.); krystyna.pogoda-sewerniak@upwr.edu.pl (K.P.-S.); 2 Department of Epizootiology and Clinic of Birds and Exotic Animals, Faculty of Veterinary Medicine, Wroclaw University of Environmental and Life Sciences, pl. Grunwaldzki 45, 50-366 Wroclaw, Poland; michal.bednarski@upwr.edu.pl

**Keywords:** iron, calves, protein-iron complex, immunology, antioxidative status

## Abstract

The objective of this study was to determine the effects of feeding protein-iron complex (PIC) on productive performance and indicators of iron metabolism, hematology parameters, antioxidant and immune status during first 35 days of a calf’s life. Preparation of the complex involved enzymatic hydrolysis of milk casein (serine protease from *Yarrowia lipolytica* yeast). Iron chloride was then added to the hydrolyzate and lyophilizate. Calves were divided into treated groups: LFe (low iron dose) 10 g/day calf of protein-iron complex, HFe (height iron dose) 20 g/day calf, and control group. Dietary supplements containing the lower dose of concentrate had a significant positive effect on iron metabolism, while the higher dose of concentrate resulted in increase of total iron binding capacity (TIBC), saturation of transferrin and decrease of and unsaturated iron binding capacity (UIBC), which suggest iron overload. Additionally, treatment with the lower dose of iron remarkably increased the antioxidant parameters, mainly total antioxidant (TAS) and glutathione peroxidase activity (GPx). Higher doses of PIC were related to lower total antioxidant status. IgG, IgM, insulin, glucose, TNFα and IGF-1 concentration did not change significantly in either group after supplementation. In practice, the use of protein-iron complex concentrate requires taking into account the iron content in milk replacers and other feedstuffs.

## 1. Introduction

Iron plays an important role in the organisms of humans and animals, and its deficiency leads to numerous health conditions. The iron requirement of animals varies according to the age, sex and condition of the organism [1,2]. Young animals in the early stages of life are most susceptible to iron deficiency, however neonates do have some iron reserves in their body. Moreover, in the case of feeding calves with cow’s milk, which has low iron concentration, rapid growth rates can lead to the development of temporary iron deficiency. This condition may be exacerbated by the immaturity of molecular mechanisms of iron absorption as in other animal species [2,3,4]. Transient iron deficiency in newborn calves most commonly manifests as anemia [2]. Presently, for young calves, the problem of iron deficiency is not as widespread as for e.g., piglets. This is due to the appropriate availability of this element in commercial milk replacers [5,6]. Young calves’ iron requirements are estimated to be around 100 ppm, and are higher than in adult animals [7]. Iron supplementation has a significant effect on optimal calf growth rate, and can play a role in physical development and in hematopoiesis [5,8]. However, it should be noted that numerous studies-including Volker and Rotermund [7] above indicate that additional iron supplementation has beneficial effects on hematological parameters as well as on other parameters associated with iron metabolism [5]. Iron supplementation may play an important role in calf growth and resistance to infections [9]. Higher concentrations of Fe in the blood of calves are also associated with a higher concentration of Insulin-like Growth Factor 1 (IGF-1), a hormone that has major effects on growth and is responsible for metabolism [9,10]. On the other hand, excessive Fe content in the diet of ruminants can have toxic effects. They cause morphological damage to the intestine by increasing their permeability. High levels of iron in feed also negatively affect the absorption of other elements; this leads to oxidative stress at the cellular level, lipid peroxidation and increased gene expression of many enzymes considered to be antioxidants [10]. Cellular respiration is disturbed, and metabolic acidosis also occurs along with the associated consequences [11].

At the present time, iron supplementation is based primarily on inorganic compounds. However, these compounds can undergo oxidation and transform into insoluble forms [4,12]. For the purposes of animal nutrition and to increase the bioavailability of this element, research focuses on the use of chelates or proteinaceous iron preparations [13]. Casein proteins have very good iron binding properties, thereby decreasing their susceptibility to oxidation and therefore have high bioavailability [13,14,15]. Only biological trials can determine what dosing of iron is optimal for growth and development of animals with regards to hematological parameters, iron metabolism, antioxidant status or immune status, depending on the form of iron administration [2,5].

The objective of this study was to determine the effects of feeding protein–iron complex (PIC) on productive performance and indicators of iron metabolism, hematology parameters, antioxidant and immune status during first 35 days of a calf’s life.

## 2. Results and Discussion

**Figure 1:**
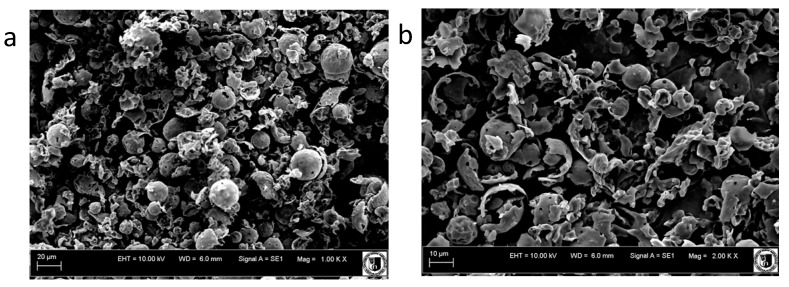

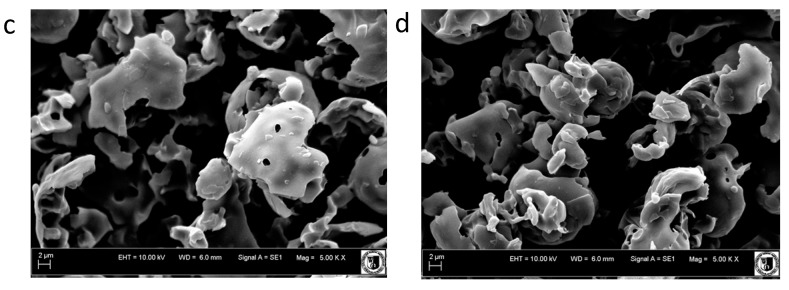
Microstructure of protein and protein-iron complex. View of the formulation at 20 and 10 μm particle size (**a**,**b**), protein fragmentation (**c**,**d**).

Casein protein concentrate is one of the most important byproducts of cheese manufacturing, and for this reason it can be used as a low-cost source of protein in food products. Iron-amino acid chelate was used for food fortification [16] as well as animal supplements [17]. Compared with amino acids, peptides are difficult to saturate with metals, although their absorption efficiency is higher. The absorption efficiency depends on the protein hydrolysis technology and the conditions of the active peptide iron chelate synthesis [18]. Analysis of the scanning electron microscopy microstructure indicates the presence of spherical particles of irregular pore size in the experimental formulation. The degree of degradation of spherical particles should be assessed as significant (Figure 1). Globules with smooth surfaces with a coarse appearance, characteristic of milk protein concentrate [19], did not occur in our study.

### 2.1. Hematological Parameters and Iron Metabolism

Iron is one of the important factors that influence growth, productivity, and animal immunity [9]. By feeding only cow’s milk to young ruminant calves, iron intake is insufficient for normal erythropoiesis during the first month of life [8], which is manifested by reduced blood parameters, such as red blood cell (RBC), hematocrit (HCT) and hemoglobin (HGB) [20]. Volker and Rotermund [7] reported that oral administration of 100 mg iron per day, prevented anemia in calves. In turn, Mohri et al. [5] showed that higher of doses of 150 mg iron per day are required by growing calves. Generally, the iron requirements of these young ruminants are higher than those of mature ones and thought to be about 100–150 mg/kg of dry matter [21]. In addition, legislation in Europe-based on the concern for animal welfare-mandates the provision of solid feed to milk-fed veal calves [22].

Hematological examinations are a basic indicator of iron metabolism and hematopoiesis. In our study, iron supplementation did not significantly increase RBC and HGB concentration in comparison to the control group (Table 1). On the other hand, our investigations showed a statistically significant decrease (*p* < 0.01) of mean corpuscular volume (MCV) in the LFe (low iron dose) group of calves and an increase in mean corpuscular hemoglobin concentration (MCHC) (both experimental groups). No differences in Mean corpuscular hemoglobin (MCH) were detected between the groups. Mohri et al. [5,8] did not observe statistically significant differences in these parameters between calves during the first 28 days of life that received either oral or parenteral iron supplementation. The MCV decrease is associated with a decrease in erythrocyte volume during the first days of life [23], but curiously, Miltenburg et al. [24] reported that MCV was correlated with the administration of iron, which does not correspond to our results. In all studies, the MCV decreased in all groups, but the decrease was fastest in the groups where the PIC was administered.

The concentration of Fe in the blood of young calves can vary by a wide range, from less than 10 μmol/L to as high as 30 μmol/L [2,25]. Moreover, over the first few days of life a progressive reduction in serum Fe concentration occurs, thus many authors point to the need for supplementation [2,20]. Primary iron deficiency is associated with a decrease of this trace element concentration in blood serum and elevated total iron binding capacity (TIBC) and unsaturated iron binding capacity (UIBC) [6]. In practice, iron deficiencies are not present when milk replacers are used, due to their high iron content. However, additional iron supplements may play an important role in calves’ growth and resistance to infections [9]. For economic reasons, inorganic forms of Fe are generally used (iron sulfate). Current technology is heading towards the use of other iron compounds that are more readily absorbed, such as chelates or protein iron complex. Studies on monogastric animals indicated that the utilization of iron-amino acid chelate is nearly twice as efficient as Fe-sulfate [17].

In the current study, the highest serum iron concentrations were observed after administering a lower dose of PIC (Table 2). No statistically significant differences were observed in mean Fe concentration between groups for the entire study period, but there were differences in the ages of the animals at which blood samples were collected. In the LFe group the increase in Fe concentration was linear, whereas in the HFe (height iron dose) group Fe concentration only increased after the first week of PIC supplementation. The UIBC showed similar dependencies. The formulations used did not cause significant differences in TIBC and transferrin saturation. At a higher dose, especially after 2 weeks of supplementation, a rising tendency could be seen in these parameters. Transferrin concentration was significantly increased in both experimental groups and, over the course of the experiment, the increase was much higher in the HFe group than in the lower dose group. The highest transferrin concentrations were observed in calves on their 28th day of life. In the HFe group, apart from growth (*p* < 0.01) of transferrin concentration, the TIBC value increased as did the percentage of transferrin iron saturation. These results indicate some iron overload (HFe group), since virtually all circulating iron in the body is bound to transferrin [26]. However, mechanisms for the removal of Fe from the body were presumably sufficiently efficient that, despite such a significant increase in transferrin, Fe concentration was similar to the control group.

When compared with other rearing methods, the use of milk replacer leads to higher Fe values in calves and saturation of transferrin, and significantly lower TIBC [27]. In our study, the highest iron concentrations occurred in the LFe group (which received a lower PIC dose), whereas no significant differences were observed between the control group and the group with the highest PIC. Similar results as the LFe group were obtained by Mohri et al. [5]. However, the results from the HFe group are not consistent with the expectation that a higher iron dose in feed correlates with higher iron concentrations as Mohri et al. [5] demonstrated at similar doses of iron. Regardless, the results are not statistically significant. A similar tendency to our study was reported by Reece and Hotchkiss [27] where a slower growth of TIBC was observed in groups where iron was administered. Mohri et al. [5] observed the opposite trend: TIBC in calves receiving higher iron doses (150 mg/day) was significantly higher in comparison to the control group, and on the 28th day it was significantly lower. In this case, the changes were probably connected with forms of iron supplementation. Studies have also been conducted on administering iron parenterally. Providing Fe-dextran via this route for 2-day-old calves resulted in improved RBC parameters, increase of Fe in blood and weight gain during the first month of life in neonatal dairy calves [1]. High iron content in the diet of other species like rats resulted in decreased growth [28]. In our own studies, the amount of Fe contained in PIC did not significantly affect the rate of body weight gain.

Studies on animal models demonstrate that iron transporter proteins in the duodenum, liver and spleen are differentially regulated during developmental iron deficiency, and that early-life iron deficiency may cause long-term abnormalities in iron recycling from the spleen [29]. A strong negative correlation was found between Fe concentration and duodenal DMT1 (Fe duodenal divalent metal transporter-1) expression in high Fe rats, suggesting that the high body Fe stores were signaling reductions in intestinal Fe transport [30].

### 2.2. Antioxidation Status, Biochemical and Immunologial Parameters

The Ganz and Nemeth [31] study suggests that excessive iron intake can lead to the production of free radicals and expose sensitive tissues to oxidative stress. The total antioxidant (TAS) value was significantly different between the groups (Table 3). The lowest mean for the entire study period was recorded in the HFe group, whereas the highest was in the control group. Statistically significant differences (*p* < 0.01) were found between the control group and the HFe group, and between the LFe group and the HFe group. These differences were apparent at the ages of 14, 28 and 35 days. Different dependencies were exhibited in glutathione peroxidase activity (GPx). The highest mean GPx activity was observed in the LFe group. At the beginning of the study, GPx activity was aligned between the groups. In the control group, the activity of this enzyme underwent a systematic reduction. Similar trends occurred in the experimental groups, albeit in the LFe group the reduction of activity was low. In the HFe group the activity decreased markedly, and the change was most apparent after one week of supplementation.

Total antioxidant assay allows an integrated antioxidant system to be evaluated, which includes all biological components that exhibit activity in realm of preventing excessive oxidation [32]. On the other hand, the determination of the major antioxidant enzymes superoxide dismutase (SOD) and GPx is aimed at evaluating the activity of intracellular antioxidants [33]. In the study conducted, on the last day of administering iron-containing compounds, the serum of experimental calves was lower in TAS, which may be attributable to a lower supply of free radicals.

The applied PIC was not confirmed to have any influence on SOD activity. However, there was a higher SOD activity in the HFe group compared to the LFe group. Iron overload in rats leads to a significant increase in catalase and SOD activity [34]. In the HFe group, higher SOD activity in the HFe group, which catalyzes the peroxide superoxide conversion reaction to hydrogen peroxide, may indicate a higher level of oxidative stress. Similar dependencies in cattle were found in other studies [35].

Long-term use of mineral blocks significantly increased serum levels of Fe, Mn, and Se, decreased the level of MDA, and increased GSH activity [36]. However, it is worth paying attention to the supply of selenium, which plays an important role in antioxidative processes. GSH-Px activity in the liver and heart was not affected by dietary Fe concentration [10], despite the administration of high doses of iron (750 mg of supplemental Fe/kg of dry matter).

No differences were found between groups regarding serum insulin and glucose levels, although at higher levels the functional additive that was used resulted in lower mean glucose and higher insulin levels. There were statistically significant (*p* < 0.01) differences in glucose concentration between the ages of the animals. In the HFe group the glucose concentration was significantly reduced, while in the control group and LFe the changes were not pronounced. In rat studies it was shown that glucose metabolism in adipose tissue appears to be affected by combination of: iron deficiency, excess through-interaction with adipocyte differentiation, tissue hyperplasia and hypertrophy, release of adipokines, lipid synthesis, and lipolysis [37]. Differences in glucose uptake in calves are largely due to the type of diet used. With milk replacers (MR) feed vs. colostrum or milk, insufficient glucose uptake was observed [38]. IGF-1 is a pleiotropic hormone exerting mitogenic and anti-apoptotic effects [39]. It is mainly produced in the liver, but can be mediated through the provision of energy, nutrients, minerals and vitamins, but also through the effects of non-nutritive factors [40]. In the case of calves born prematurely, there is a reduced thyroid status, as well as reduced IGF-1 concentrations in plasma [41]. Research on animal models and humans has revealed an important relationship between iron and IGF-1 [42,43]. Chronic iron deficiency leads to a decrease in hemoglobin in the blood, which results in a decrease in IGF-1 secretion in the liver [43]. Induced gestational-neonatal iron deficiency in rats indicates that early postnatal iron treatment of gestational iron deficiency reactivates the IGF system and promotes neurogenesis and differentiation in the hippocampus during a critical developmental period [44]. A reduction of IGF-1 secretion is also caused by iron overload [43].

Generally, calves exposed to iron deficiency anemia presented with a significant (*p* < 0.01) reduction in serum IGF-1 compared to calves with optimal iron concentration during the neonatal period [2]. Moderate IGF-1 serum concentrations were unaffected by dietary treatment (Table 3). A four-week supplementation regime (age 35 days) resulted in LFe iron level of 19.36, while in group III 16.59 μmol/L, with IGF-1 concentrations of 50.29 and 38.44 ng/mL, respectively. In the control group, Fe concentration was 17.80 μmol/L, with IGF-1 concentration of 43.26 ng/mL. These data indicate the optimal normalization of Fe concentration in the LFe group.

There exist dependencies between the level of feeding and the concentration of IGF-1 in the blood. Calves that are fed high protein and energy have a higher plasma concentration of IGF-1 than calves fed with a moderate or low diet [45]. In our studies, despite the fact that the supplementation was small, these dependencies were only confirmed in the LFe group. Concentrations of IGF-1 were affected by the amount of untreated calf milk replacer fed and were consistently higher in calves that were fed additional milk replacer [46]. Long-term iron supplementation in young dairy cattle results in an increase in IgG and IGF-1 concentration [47]. On the first day of our study, the IgG concentrations were measured and found to be equal in the groups, indicating that the passive immunity was adequate. Neither the dietary group nor the timing of blood sampling had any effect on serum IgG or IgM concentrations.

Infections can initiate an immune response, with concomitant production of cytokines such as tumor necrosis factor-α (TNF-α), interferon-γ (IFN-γ), interleukin-6 (IL-6), interleukin-10 (IL-10), and interleukin-1β (IL-1β) [46,48]. In practice, gastrointestinal infections are quite common [49]. This response is amplified by shipping stress followed by exposure to environmental pathogens. Animal feed has some effect on TNF-α concentration. Calves that had been fed once secreted less TNF-α in lipopolysaccharide stimulated whole blood cultures at 45 days of age compared with twice-fed calves, and these concentrations tended to persist through the immediate postweaning period [50]. A significant reduction in TNF-α was observed in adult ruminants that were fed with amino-acid-protected supplementation [51]. In our own studies, serum concentrations of TNF-α were unaffected by treatment (Table 3). In all of the studied calves, a decreasing trend was observed at day 14 in the serum TNF-α concentrations, followed by a subsequent increase. Clinical examinations as well as measured changes in IGF1; TNF-α and SOD activity were not significant, indicating that the calves did not exhibit inflammatory conditions that affected the formation of reactive oxygen species. Thus, an objective assessment of the impact of the dietary supplement could be made.

Total antioxidant activity decreased markedly after four weeks of supplementation of protein-iron complex, which may be attributable to a lower supply of free radicals or which suggests a decreased ability of preventing excessive oxidation. On the other hand, excessive iron intake can lead to the production of free radicals. The lower dose of protein-iron complex had a beneficial effect on the antioxidant status and the concentration of IGF-1 and iron in the blood at 35 days of age in comparison to the group with the higher dose of PIC.

## 3. Materials and Methods

### 3.1. Statement of Ethics

Studies have been conducted in accordance with the European Animal Welfare Guidelines and GLP. All the calves were handled in accordance with the regulations of the Polish Council on Animal Care and all the procedures for this trial were approved by the 2nd Local Ethical Committee for Experiments on Animals in Wroclaw (No. 63/2013).

### 3.2. Animals and Treatments

The study was carried on 30 Polish Holstein Friesian calves of the black-and-white variety. The animals were put into randomized groups, taking into account the age (7 days of age), body weight (ca. 40 ± 1.65 kg) and sex (50% females and 50% males in each group). The calves received a commercial milk replacer (Polmass, Bydgoszcz, Poland), and ad libitum granulated feed (Josera, Nowy Tomysl, Poland). Clean water was available at all times. Milk replacers were fed at 7:00 and 14:30 daily. During the first 21 days the calves received 6 L/day of MR, and then 8 L/day.

The calves were divided into: control group, fed with standard milk replacer (*n* = 10); experimental group (LFe), receiving a PIC additive in milk replacer at 10 g/day (*n* = 10); and HFe group, receiving PIC at 20 g/day (*n* = 10). The milk replacer formula contained 100 mg of iron in the form of iron sulfate; calves received an additional 27 mg (LFe) or 54 mg (HFe) of iron contained in protein-iron complex (PIC). Body mass of the calves was measured before morning feeding at 7, 14, 28, and 35 days of age. Dry matter intake was controlled in this study.

### 3.3. Process of Obtaining Protein-Iron Complex

Isoelectric casein was suspended in water and conditioned for 10 min at 60 °C, then cooled to 35 °C and basified to pH 8.0 using 5 mol NaOH (Merck, Darmstadt, Germany). In the next step, serine protease (enzyme preparation from *Yarrowia lipolytica* yeast) was introduced. Enzymatic hydrolysis was carried out for 3 h at 35 °C using 120 rpm stirring. The hydrolysis was interrupted by thermal deactivation of the enzyme at 80 °C for 20 min, then the hydrolyzate was cooled to 2 °C and a solution of Sigma-Aldrich iron (II) chloride (Buchs, Switzerland) was introduced. To a final concentration of 0.001 mol. binding was carried out under cooling for 12 h and then the resulting formulation was spray dried. The detailed process for obtaining the protein-iron complex is the subject of a patent application. The Fe contents were analyzed using an atomic absorption spectrophotometer Varian SpectrAA 220 (Agilent Technologies Inc., Palo Alto, Santa Clara, CA, USA) [52]. This analysis was used to determine the dose of PIC in treatment groups.

### 3.4. Scanning Electron Microscopy

The microstructure of protein-iron complex was examined by scanning electron microscopy using a Leo Zeiss 435 VP (Oberkochen, Germany), operating at 10 kV. Technical preparation of the samples included standard procedures for this type of microscopy, which were performed at Wroclaw University of Environmental and Life Sciences. Micrographs were recorded.

### 3.5. Clinical Observations and Sampling Procedures

During the study the calves were placed under clinical observation. Vitality, dehydration and fecal consistency were determined on the basis of a clinical trial and follow-up at 7, 14, 28, and 35 days of age. Blood samples were collected from all calves from the external jugular vein (*vena jugularis externa*) at 7, 14, 28, and 35 days of age. Seven days of age was treated as the starting point of the PIC (supplementation) experiment. For each sample, blood was drawn into a tube containing K2EDTA, a tube containing sodium heparin, and into a tube without anticoagulant (Sarstedt, Warsaw, Poland). The blood samples for serum and plasma were centrifuged at 3000× *g* for 10 min at a room temperature (2 h from collection), and the serum samples were frozen (−20 °C) until the analysis.

### 3.6. Laboratory Analyses

Blood tests were performed to assess the effects of hematological, biochemical and immunological parameters. Analysis of hematological parameters was performed using an ABC Vet analyzer (Horiba ABX, Montpellier, France), which recorded parameters that included: red blood cells (RBC), white blood cells (WBC), hemoglobin (HGB), hematocrit (Hct), mean corpuscular volume (MCV), mean corpuscular hemoglobin (MCH), platelets (PLT) and mean corpuscular hemoglobin concentration (MCHC).

Iron was measured in serum by photometric test using Ferene Horiba ABX (Montpellier, France). Serum Unsaturated Iron Binding Capacity (UIBC) was determined by using a photometric method with ferrosine using reagents from Pointe Scientific (Canton, MI, USA). Serum Total Iron Binding Capacity (TIBC) was determined by using a photometric method, precipitating Fe^3+^ with calcium carbonate, using a BioMaxima (Lublin, Poland) precipitant, followed by a Horiba ABX (Montpellier, France) reagent for iron determination. Transferrin serum was labeled with the Bethyl (Montgomery, AL, USA) Bovine Transferrin ELISA Kit for testing immuno-enzymes. Transferrin saturation (TS) was calculated by TS = [Iron (μmol/L)]/[TIBC (μmol/L)] × 100 [L].

The laboratory analyses of blood serum were done using Pentra 400 biochemical analyzer Horiba ABX (Montpellier, France). The following parameters were estimated:

- glucose by oxidase method, reagents HORIBA ABX (Montpellier, France);
- glutathione peroxidase activity (GPx) by enzymatic method, Randox reagents Ransel RS (Crumlin, UK). The parameters determining the anti-oxidative status were also determined:
- Total antioxidant capacity (TAS) in serum by colorimetric method based on ABTS (2,2′-azine-di-[3-ethylbenzothiazoline sulfate]) method with peroxidase,
- glutathione peroxidase (GPx) in whole blood using enzymatic method,
- superoxide dismutase (SOD) in erythrocytes by the spectrophotometric, consisting of reaction with 2-(4-iodophenyl-3-(4-nitrophenol)-5-phenyltetrazoline chloride (I.N.T.)

These analyses were performed with the Pentra 400 biochemical analyzer by Horiba ABX (Montpellier, France), using reagents from Randox (Crumlin, Dunlin, Ireland).

Immunological parameters were determined in serum. Serum IgG immunoglobulin was assayed using a Bethyl Bovine IgG ELISA Kit. Serum IgM immunoglobulin was determined by Bovine IgM ELISA Kit, Bethyl (Montgomery, AL, USA). Tumor Necrosis Factor (TNF-α) was assayed in serum by the Bovine Tumor necrosis factor ELISA Kit from MyBio Source (San Diego, CA, USA). Serum IL-6 interleukin was assayed by the Bovine Interleukin-6 ELISA Kit immunoassay from MyBio Source (San Diego, CA, USA). Insulin-like growth factor 1 (IGF1) was assayed in serum using an enzyme-linked immunosorbent assay (IGF-1) ELISA Kit from MyBio Source (San Diego, CA, USA). Concentration of insulin in serum was determined by the ELISA method using the BioSource INS-EASIA Kit (BioSource Europe SA, Louvain-la-Neuve, Belgium). The above measurements were conducted using a Synergy fluorescence, luminescence and absorbance reader from BioTek Instruments (Winooski, VT, USA).

### 3.7. Statistical Analysis

Results obtained were subjected to statistical analysis using STATISTICA 10.0 software (Statistica, Tulsa, OK, USA). Data were analyzed using a general linear model for repeated measures ANOVA with dietary treatments (D) and sampling time (T) as fixed effects and their interactions (D × T) according to the model:

*Y*_ijk_ = μ + α_i_ + β_j_ + αβ_ij_ + ε_ijk_

where *Y*_ijk_ is the dependent variable; μ is the overall mean; α_i_-group (three groups); β_j_-series of blood tests (1, 2, 3, 4); αβ_ij_-group effect x series of tests; ε*_ijk_*-random residual error.

Differences between treatment group means were analyzed for significance (*p* < 0.05) using the Duncan test. Production and health data were subjected to the nonparametric Wilcoxon test. The data are presented as average values and accompanied by standard error of the means.

## 4. Conclusions

The study has shown that the iron contained in the milk replacer feed only satisfies the basic iron requirements for erythropoiesis. Only the lower dose of protein-iron complex had a positive effect on the immune and antioxidant status. Further studies should focus on iron concentrations in the liver, as well as hepcidin-ferroportin axis and cytotoxicity, after adding PIC to the diets of calves.

## Figures and Tables

**Table 1:** Mean values of hematological parameters in calf blood.

Item	Treatment			SEM	*p*-Value ^1^		
	Control	Low Iron Dose (LFe)	Height Iron Dose (HFe)		D	T	D × T
WBC (G/L)	9.95	9.48	7.93	0.28	<0.01	0.11	0.88
RBC (T/L)	8.04	8.02	8.14	0.10	0.87	0.26	0.92
HGB (mmol/L)	6.01	6.21	6.25	0.09	0.60	0.25	0.79
HCT (L/L)	0.31	0.27	0.32	0.07	<0.01	0.37	0.41
PLT (G/L)	757.47	770.93	822.34	30.22	0.64	<0.01	0.89
MCV (fl)	38.39	33.64	39.29	0.28	<0.01	<0.01	0.14
MCH (fmol)	0.77	0.76	0.77	0.04	0.40	0.12	0.65
MCHC (mmol/L)	19.36	23.00	19.53	0.14	<0.01	<0.01	0.54

^1^ Significant effect of experimental diet (D), time on diet (T), and their interaction (D × T); SEM-standard error of the means.

**Table 2:** Mean value of iron metabolism parameters.

Item	Treatment			SEM	*p*-Value ^1^		
	Control	Low Iron Dose (LFe)	Height Iron Dose (HFe)		D	T	D × T
Iron (µmol/L)	15.06	16.59	14.70	0.57	0.35	0.01	0.88
UIBC (µmol/L)	6.68	5.47	6.15	0.46	0.62	<0.01	0.98
TIBC (µmol/L)	19.99	18.30	18.72	0.56	0.76	0.11	0.52
Transferrin saturation (%)	70.484	73.77	79.10	0.94	0.03	0.02	0.99
Transferrin (mg/mL)	2.041	3.59	5.53	0.42	<0.01	<0.01	0.01

^1^ Significant effect of experimental diet (D), time on diet (T), and their interaction (D × T); SEM-standard error of the means.

**Table 3:** TAS, GPx, SOD and concentration of selected biochemical and immunological parameters in calf blood.

Item	Treatment			SEM	*p*-Value ^1^		
	Control	Low Iron Dose (LFe)	Height Iron Dose (HFe)		D	T	D × T
TAS (mmol/L)	1.15	0.91	0.86	0.22	<0.01	0.79	0.13
GPx (U/L)	59,716.22	60,833.10	50,422.23	324.11	0.01	0.01	0.88
SOD (U/mL)	1168.6	1041.8	1249.4	30.45	0.31	0.36	0.99
Insulin (ng/mL)	0.496	0.589	0.620	0.03	0.12	0.18	0.11
Glucose (mmol/L)	5.992	5.783	5.459	0.11	0.16	0.01	0.66
IGF-1 (ng/mL)	45.251	50.761	40.849	1.42	0.30	0.51	0.98
TNF-α (pg/mL)	103.76	108.79	95.891	1.14	0.49	0.15	0.71
IgG (mg/mL)	12.355	13.502	11.256	0.54	0.23	0.09	0.86
IgM (mg/mL)	0.70125	0.64937	0.78187	0.05	0.52	0.67	0.17

^1^ Significant effect of experimental diet (D), time on diet (T), and their interaction (D × T); SEM-standard error of the means.

## Abbreviations

PIC	Protein-iron complex
MR	Milk replacers
IGF-1	Insulin-like Growth Factor 1
SEM	Scanning electron microscopy
RBC	Red blood cell
WBC	White blood cell
HCT	Hematocrit
HGB	Hemoglobin
MCV	Mean corpuscular volume
MCH	Mean corpuscular hemoglobin
MCHC	Mean corpuscular hemoglobin concentration
DM	Dry matter
PLT	Platelets
TIBC	Total iron binding capacity
UIBC	Unsaturated iron binding capacity
TS	Transferrin saturation
LFe	Experimental group receiving low iron dose
HFe	Experimental group receiving height iron dose
DMT1	Duodenal divalent metal transporter-1
TAS	Total antioxidant capacity
GPx	Glutathione peroxidase activity
SOD	Superoxide dismutase
MDA	Malondialdehyde
GSH-Px	Erythrocyte glutathione peroxidase activity
TNF-α	Tumor necrosis factor-α
IFN-γ	Interferon-γ
IL	Interleukin
IgG	Immunoglobulin G
IgM	Immunoglobulin M
